# Ferumoxytol for iron deficiency anemia in patients undergoing hemodialysis. The FACT randomized controlled trial 

**DOI:** 10.5414/CN109512

**Published:** 2019-02-25

**Authors:** Iain C. Macdougall, William E. Strauss, Naomi V. Dahl, Kristine Bernard, Zhu Li

**Affiliations:** 1 Department of Renal Medicine, King’s College Hospital, London, UK, and; 2 AMAG Pharmaceuticals, Inc., Waltham, MA, USA

**Keywords:** anemia, chronic kidney disease, dialysis, iron overload, iron sucrose

## Abstract

Background: Patients with chronic kidney disease (CKD) undergoing dialysis often require intravenous iron for iron deficiency anemia (IDA). Materials and methods: The Ferumoxytol for Anemia of CKD Trial (FACT), a randomized, multicenter, open-label, phase 4 study, compared the long-term safety and efficacy of ferumoxytol with iron sucrose for the treatment of IDA in patients with CKD undergoing hemodialysis. Patients with IDA and CKD undergoing hemodialysis were randomized 2:1 to ferumoxytol 1.02 g (2 × 510 mg) or iron sucrose 1.0 g (10 × 100 mg) for a 5-week treatment period (TP). Over 11 months, patients underwent additional 5-week TPs whenever IDA (hemoglobin < 11.5 g/dL and transferrin saturation < 30%) was detected. The primary efficacy endpoint was mean change in hemoglobin from baseline to week 5 for each TP. Adverse events were recorded during the study. Results: Overall, 293 patients received ferumoxytol (n = 196) or iron sucrose (n = 97). Ferumoxytol was noninferior to iron sucrose regarding hemoglobin change from baseline to week 5. The mean change in hemoglobin in the ferumoxytol and iron sucrose groups was 0.5 and 0.4 g/dL, respectively, in TP 1 (least-squares mean difference, 0.13; 95% confidence interval, –0.11 to 0.36) and 0.6 and 0.3 g/dL, respectively, in TP 2 (0.30; 0.06 – 0.55). Treatment-related and serious adverse events were similar in both groups; no new safety signals emerged. Conclusion: Long-term administration of ferumoxytol has noninferior efficacy and a similar safety profile to iron sucrose when used to treat IDA in patients with CKD undergoing hemodialysis.

## Introduction 

Iron deficiency anemia (IDA) is a common condition among patients with chronic kidney disease (CKD) undergoing hemodialysis [1]. The etiology of IDA among patients with CKD is multifactorial, including recurrent blood loss during hemodialysis and reduced gastrointestinal absorption of dietary iron due to increased hepcidin activity [1, 2]. The resulting iron deficiency is compounded by an increased demand for iron secondary to erythropoiesis-stimulating agent (ESA) use, which cannot usually be met by oral iron supplementation; therefore, intravenous (IV) iron has become the standard of care for these patients [3], with a mean dosage of ~ 3.3 to 3.4 g per year [4]. 

IV iron may be administered as either a single large dose or several smaller doses, depending on the preparation used. Treatment guidelines suggest that IV iron can be administered on either a maintenance therapy schedule, where iron is administered at regular intervals to maintain iron levels, or as needed, when indices of iron status indicate that iron stores are being depleted [5]. However, despite widespread use of IV iron, there are remarkably few randomized controlled trials (RCTs) directly comparing different IV iron preparations for efficacy or safety. Most studies have been noncomparative, of short duration (< 3 months), or involved small numbers of patients (n < 60) [6, 7, 8, 9, 10, 11, 12, 13, 14]. Comparative data evaluating the efficacy and safety of ferumoxytol versus other IV iron preparations among patients with CKD are limited to one short-term study [11], thus highlighting the need for long-term, repeat-dosing, head-to-head studies of these agents. 

The Ferumoxytol for Anemia of CKD Trial (FACT) evaluated IV ferumoxytol versus iron sucrose in the treatment of IDA among patients with CKD undergoing hemodialysis over a 1-year period [15]. The primary objectives of the FACT study were to demonstrate noninferiority of ferumoxytol compared with iron sucrose at increasing hemoglobin levels for IDA treatment, as well as safety, when used as needed in patients with CKD undergoing hemodialysis over a 1-year period. 

## Materials and methods 

### Study design and patient population 

FACT was a phase 4, randomized, open-label, active-controlled study (ClinicalTrials.gov identifier: NCT01227616 (registered October 22, 2010)) conducted at 35 sites in the United States (US), Canada, and the United Kingdom from August 2013 to February 2016. The study was conducted in accordance with the Declaration of Helsinki and Good Clinical Practice guidelines, and the protocol was submitted to and approved by the appropriate investigational review boards. All patients provided written informed consent before study participation. Details regarding the study design are reported elsewhere [15]. Adults (aged ≥ 18 years) with CKD undergoing hemodialysis for ≥ 3 months before screening with IDA (defined as hemoglobin levels at screening of < 11.5 g/dL and transferrin saturation (TSAT) < 30%) were included. Patients were excluded if serum ferritin was > 800 ng/mL. 

### Treatment regimen and concomitant therapy 

**Figure 1.**
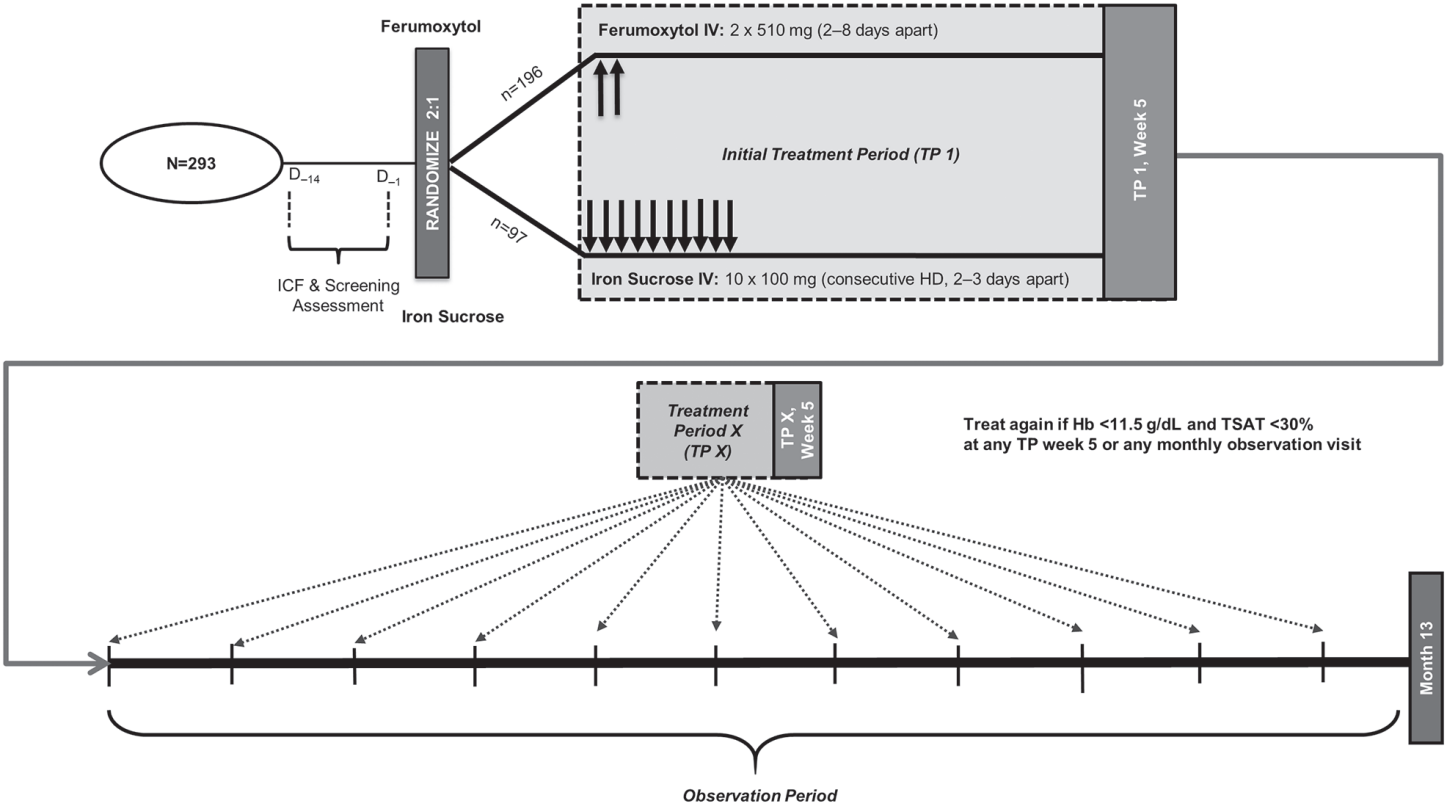
Study design and treatment. D = day; Hb = hemoglobin; HD = hemodialysis; ICF = informed consent form; IV = intravenous; TP = treatment period; TSAT = transferrin saturation.

Patients were randomized in a 2:1 ratio to receive ferumoxytol or iron sucrose for an initial 5-week treatment period (TP) (Figure 1). Ferumoxytol was administered as follows: two IV injections of ferumoxytol 510 mg for a total of 17 mL over ~ 1 minute at a rate not to exceed 1 mL/s, or as a diluted IV infusion over ≥ 15 minutes on day 1; the second dose was administered 5 ± 3 days after the first dose, for a total cumulative dose of 1.02 g. The change in the ferumoxytol administration from injection to infusion occurred during the latter stages of the study; therefore, most patients received ferumoxytol by injection. Iron sucrose was administered as an IV injection or infusion of 100 mg on day 1 and during the following nine consecutive hemodialysis sessions for a total cumulative dose of 1.0 g. Patients not previously exposed to iron sucrose received a test dose before the first study administration. 

During the subsequent 11-month observation period, patients who had persistent or recurrent IDA (hemoglobin < 11.5 g/dL and TSAT < 30%) at any monthly observation visit or at the final visit of any TP underwent another 5-week TP with their original randomized treatment (Figure 1). After the initial assigned iron treatment, any patient receiving a different IV iron treatment from their initial randomization assignment was withdrawn from the study. Patients receiving ESA therapy were not to undergo any dose modifications during any TP except when required for safety reasons. The total ESA dose administered was recorded monthly. For analysis, darbepoetin-α and methoxy polyethylene glycol-epoetin-β were converted to equivalent epoetin-α doses according to their prescribing information. 

### Study endpoints 

The primary efficacy endpoint was the mean change in hemoglobin from baseline to week 5 for each TP since it reflects the primary desired response to a course of IV iron in patients receiving treatment for IDA. Secondary efficacy endpoints for each TP were the mean change in TSAT from TP baseline to week 5 and the proportion of patients with a hemoglobin increase of ≥ 1.0 g/dL at any time between TP baseline and week 5. Exploratory efficacy endpoints included the time to subsequent treatment courses of ferumoxytol or iron sucrose; cumulative IV iron exposure for each patient; the proportion of patients requiring blood transfusion; and the proportion of patients who had a change in ESA dose. The safety analysis involved an evaluation of the adverse-event (AE) profile of ferumoxytol and iron sucrose following each TP and over the study duration, including serious AEs (SAEs); AEs leading to study drug discontinuation; all AEs; protocol-defined AEs of special interest (including hypotension and hypersensitivity reactions); and routine laboratory parameters. 

### Exploratory substudies 

Two substudies were performed: one investigating oxidative stress and the other using magnetic resonance imaging (MRI) to determine iron load. Details regarding the design of the substudies have been reported previously [15]. 

The primary objective of the oxidative stress substudy was to evaluate changes in blood biomarkers of oxidative stress/inflammation during the initial 5-week TP. The primary objective of the MRI substudy was to assess the change from baseline in cardiac iron deposition using cardiac MRI (T2*) at 6, 12, and 24 months after the first TP. Secondary objectives included the proportion of patients with a cardiac T2* of < 20 and < 10 ms, the change in liver iron concentration (LIC) by MRI T2*, and the change from baseline in ferritin, TSAT, liver and thyroid function, and blood glucose and glycated hemoglobin (HbA1c) levels at 6, 12, and 24 months after the first TP. At screening, participants in the MRI substudy underwent an initial MRI of their heart and liver and blood tests. At the 6-, 12-, and 24-month visits, patients underwent an MRI and blood tests as per the screening visit; AEs were also captured during these visits. 

### Statistical analysis 

Enrollment of 300 patients was planned, with an estimated retreatment rate of 80% (n = 240). A sample size of 240 patients provided ~ 90% power for testing the noninferiority of ferumoxytol to iron sucrose, assuming a noninferiority margin of 0.5 g/dL, a one-sided α level of 0.025, and a standard deviation (SD) of 1.13 g/dL for the primary endpoint of change from baseline in hemoglobin. The sample size of the study was based on the predicted number of patients to be retreated at the second TP; therefore, noninferiority analysis of the efficacy determination was prespecified for the first two TPs only. 

Three analysis populations were defined: (1) the safety population (all patients who had any exposure to study drug); (2) the intention-to-treat (ITT) population (all patients with any exposure to ferumoxytol or iron sucrose); and (3) the evaluable population (all patients who did not violate inclusion/exclusion criteria and had full treatment and evaluable hemoglobin data at baseline and week 5 in the initial TP). The ITT population was used for the analysis of efficacy endpoints; analysis of the primary efficacy endpoint was also performed on the evaluable population. The oxidative stress and MRI substudy analyses were conducted using data from the evaluable population. All safety analyses used data from the safety population; missing safety data were not imputed. 

## Results 

### Patients 

Of the 296 randomized patients, 293 received the study drug (ferumoxytol, n = 196; iron sucrose, n = 97) and were included in ITT and safety populations (Supplemental Figure 1). The evaluable population comprised 264 patients (ferumoxytol, n = 181; iron sucrose, n = 83), and study treatment was completed by 216 patients (73.7%) overall, with completion rates of 72.4% and 76.3% in the ferumoxytol and iron sucrose groups, respectively. In the ITT population, early discontinuation of the study drug was reported for 10 patients (5.1%) in the ferumoxytol group and 1 (1.0%) in the iron sucrose group. 

Patient demographics and baseline clinical characteristics were generally well balanced between treatment groups (Table 1). Patients had a mean (SD) age of 58.8 (14.0) years, 58.4% were male, and 50.5% were white. Most patients (93.9%) were enrolled at US study sites. 

### Efficacy 

**Primary endpoint**

**Figure 2.**
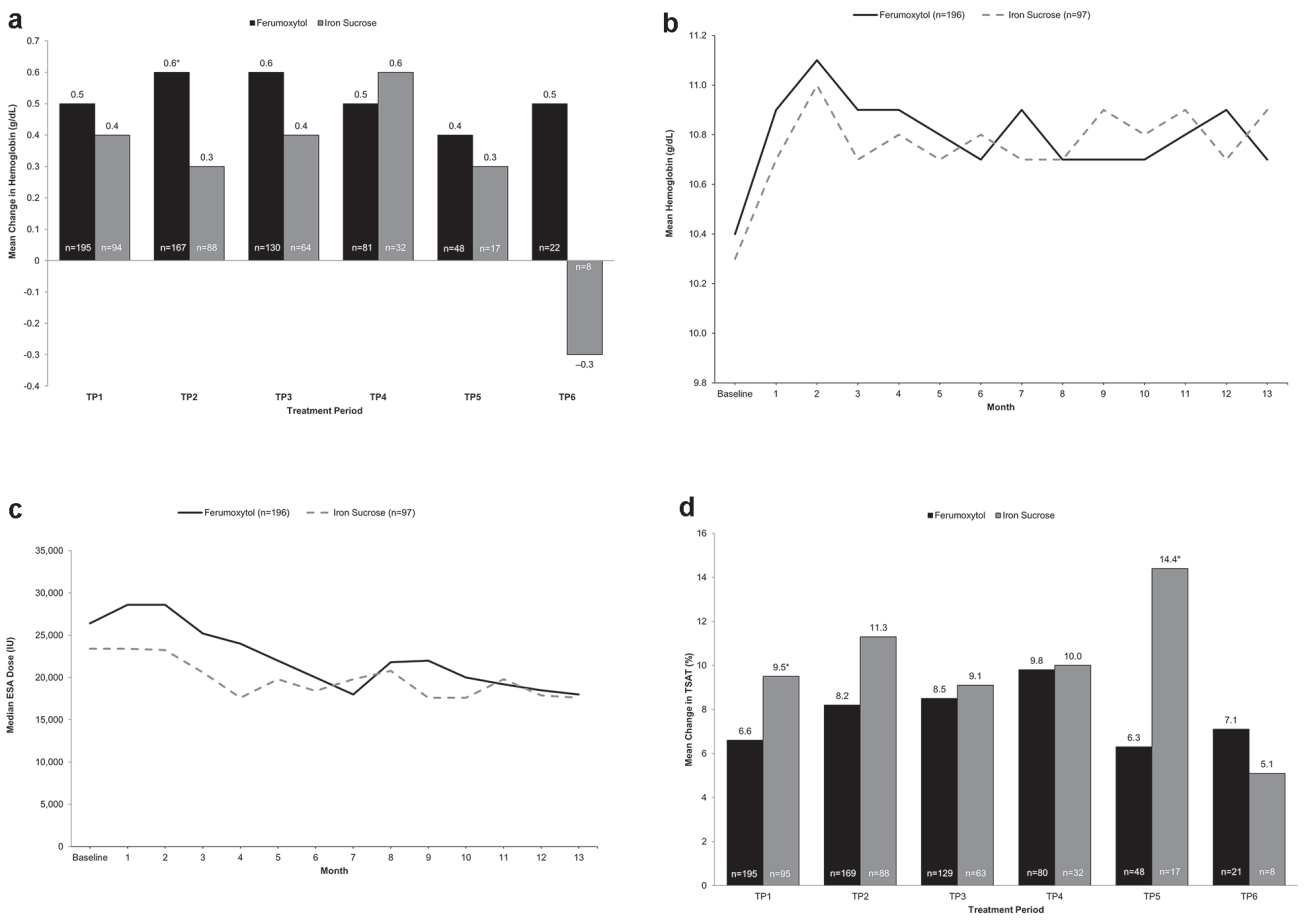
a: Mean change in hemoglobin at week 5. b: Mean hemoglobin levels by month for each treatment group over the course of the study; c: Median monthly ESA dose by treatment group over the course of the study; d: Mean change in TSAT at week 5. For Panels a and b, TP includes only those periods for which n > 5 for both treatment groups. For each TP, the number of patients treated with ferumoxytol and iron sucrose, respectively, is shown in brackets. ESA = erythropoiesis-stimulating agent; TP = treatment period; TSAT = transferrin saturation. *p < 0.05 vs. comparator.

In the ITT population, the mean change from baseline in hemoglobin at week 5 was 0.5 and 0.4 g/dL with ferumoxytol and iron sucrose, respectively, for TP 1 (least-squares mean (LSM) difference, 0.13; 95% confidence interval (CI), –0.11 to 0.36) and 0.6 and 0.3 g/dL for TP 2 (LSM difference, 0.30; 95% CI, 0.06 – 0.55) (Figure 2a). Because the lower bound of the 95% CI for the LSM differences was greater than –0.5 g/dL, the noninferiority of ferumoxytol versus iron sucrose regarding mean changes in hemoglobin levels over 5 weeks was established for the first two TPs. 

During the study, mean hemoglobin levels in both treatment groups increased by ~ 0.6 g/dL over the first 2 months of treatment, after which they generally remained stable until the end of the study (Figure 2b). 

**Additional efficacy endpoints**

ESA dosing was equivalent between the ferumoxytol and iron sucrose treatment groups, and the proportion of patients who required a change in ESA dose was similar. From baseline to month 13, decreases in median ESA doses of ~ 30% were observed with both ferumoxytol (26,400 to 18,000 IU) and iron sucrose (23,400 to 17,600 IU) (Figure 2c). 

The proportion of patients with a hemoglobin increase of ≥ 1 g/dL at any time during each 5-week TP was similar for both groups in most TPs (5 out of 6). Note; hemoglobin change was assessed only for the first 6 TPs, in which > 5 subjects in both treatment groups received study medication. 

Mean TSAT levels increased from baseline at week 5 with ferumoxytol and iron sucrose in all TPs (Figure 2d). Over the course of the study, mean TSAT levels in both treatment groups increased after 1 month of treatment and stabilized in subsequent months towards the end of the study, with mean (SD) increases from baseline at month 13 of 9.2% (12.6%) and 12.7% (14.5%) in the ferumoxytol and iron sucrose groups, respectively. 

**Exploratory endpoints**

The median time intervals between subsequent treatment courses, either between the first dose of successive TPs or between the last dose of one TP and the first dose of the next TP, were similar in both treatment groups. Over the course of the study, mean (SD) cumulative IV iron exposure was similar in both treatment groups 3,502.5 (1,759.8) mg for ferumoxytol vs. 3,117.6 (1,549.6) mg for iron sucrose), with median values of 3,060 mg for ferumoxytol and 3,000 mg for iron sucrose. 

The proportion of patients who required a blood transfusion was similar between the ferumoxytol (9.2% (n = 18/196)) and iron sucrose groups (8.2% (n = 8/97)), as was the mean number of transfusions (1.9 vs. 1.4, respectively). 

### Safety 

Overall, treatment-emergent AEs (TEAEs) were reported by 239 of 293 patients (81.6%). The incidences of TEAEs, treatment-related TEAEs, and SAEs were similar in the ferumoxytol and iron sucrose groups (Table 2). None of the serious TEAEs or post-treatment deaths were considered by the investigators to be study drug related. 

Protocol-defined AEs of special interest, which included moderate to severe hypotension and hypersensitivity reactions, occurred at a higher incidence with iron sucrose (26.8%) than with ferumoxytol (12.8%) (Table 2); however, the types of AEs were consistent with those known to be associated with IV iron products, and no new safety signals were identified. 

Permanent discontinuation of the study drug because of an AE occurred in 8 patients (4.1%) in the ferumoxytol group and no patients in the iron sucrose group (Table 2). The AEs for 2 of the 8 patients were considered to be study drug related (pruritus and urticaria in one and myalgia in the other). Study discontinuation because of AEs occurred in 20 patients (10.2%) in the ferumoxytol group and 7 patients (7.2%) in the iron sucrose group; this is inclusive of the permanent discontinuation occurrence in the ferumoxytol group deemed study drug related (pruritus and urticaria). 

### Exploratory substudies 

**Oxidative stress substudy**

The oxidative stress substudy enrolled 124 patients from the main study; of these, 96 were included in the substudy evaluable population (patients who had at least one pair of baseline and postbaseline values of any of the collected markers) for analysis. Biomarkers assessed included protein carbonyl content, monocyte chemoattractant protein-1, neutrophil gelatinase-associated lipocalin, and high-sensitivity interleukin-6. In the initial 5-week TP during our study, unlike previous single-center studies, the mean levels of various oxidative stress/inflammatory biomarkers showed no significant changes from baseline with either ferumoxytol or iron sucrose (Supplemental Table 1). 

**MRI substudy**

The MRI substudy was terminated early because of low patient enrollment related to testing logistics; there was also marked attrition of enrolled patients after the 12-month time point. At the time of study termination, 46 patients (ferumoxytol, n = 26; iron sucrose, n = 20) were enrolled in the substudy and 25 patients (ferumoxytol, n = 15; iron sucrose, n = 10) were evaluable for the MRI substudy (had a baseline MRI and at least one follow-up measurement). Because of early substudy termination, patient data were only available for the 6- and 12-month time points (not for the full 2 years). 

**Cardiac iron content**

At 6 and 12 months, mean cardiac iron content (primary substudy endpoint) was essentially unchanged in both the ferumoxytol and iron sucrose groups (Supplemental Table 2). Mean (SD) changes in cardiac T2* at 6 and 12 months were –1.90 (9.32) and –2.74 (7.00) ms, respectively, for ferumoxytol and –1.04 (6.46) and 0.56 (6.55) ms, respectively, for iron sucrose. Changes that resulted in cardiac T2* values within the normal range (i.e., > 20 ms) were considered not to be clinically significant. Cardiac T2* values < 20 or < 10 ms were not detected in either treatment group at either time point. 

### Hepatic iron content 

Increases from baseline in LIC were detected in both treatment groups at 6 and 12 months (Supplemental Table 2). Increases in LIC were higher with ferumoxytol than with iron sucrose, with mean (SD) changes in hepatic T2* at 6 and 12 months of 13.33 (6.96) and 14.21 (9.41) ms, respectively, for ferumoxytol and 4.05 (2.03) and 2.95 (2.13) ms, respectively, for iron sucrose. As both groups received statistically-identical total amounts of iron and had similar responses in hemoglobin and TSAT levels, this three-fold difference in LIC is considered to be an artifact caused by the paramagnetic properties of ferumoxytol. Systematic overestimation of hepatic iron values by MRI has been previously described in healthy volunteers receiving ferumoxytol for diagnostic imaging [16]. 

In addition to the results provided here, there were no clinically significant differences in any of the laboratory parameters assessed in the substudy, including liver function tests, thyroid function tests, and HbA1c (data not shown). 

## Discussion 

Administration of ferumoxytol in this study provided changes from baseline in mean hemoglobin that were comparable with those seen with iron sucrose after each TP, with similar dose requirements of ESA therapy; the efficacy of ferumoxytol met the criteria for noninferiority versus iron sucrose. Mean hemoglobin and TSAT levels increased after 5 weeks in both treatment groups, and these increases were maintained for 1 year by “as-needed” administration of the two iron regimens. In addition, the maintenance of stable hemoglobin levels could not be attributed to changes in ESA dose, as ESA use decreased to a similar extent in both groups. TEAEs and treatment-related TEAEs occurred at a similar rate in both treatment groups, and there were no treatment-related SAEs or deaths reported. The proportion of patients experiencing an SAE in the FACT study was similar among both treatment groups. 

Compared with previous RCTs in this patient population, the number of TEAEs in the FACT study appears to be higher, almost certainly because of the longer follow-up duration (13 months). In a previous open-label RCT of ferumoxytol versus oral iron among patients with CKD undergoing hemodialysis, SAEs were experienced by 12.7% of ferumoxytol recipients over a 5-week period [17]. Although the AE profiles of ferumoxytol and iron sucrose were similar, patients receiving iron sucrose had a higher incidence of AEs of special interest. This finding was driven by a higher incidence of an acute drop in systolic blood pressure of ≥ 30% with iron sucrose (26%) versus ferumoxytol (10%), although it should be noted that AEs leading to permanent drug discontinuation occurred more often with ferumoxytol (4.1%) than with iron sucrose (0.0%). The higher occurrence of hypotension observed with iron sucrose was presumably related to the fact that 10 administrations of iron sucrose were required to deliver a course of IV iron vs. 2 with ferumoxytol. Cumulative iron exposure did not differ by treatment regimen (median doses of 3,060 mg for ferumoxytol and 3,000 mg for iron sucrose) and was consistent with expected annual use from the literature [4]. 

Concerns have been raised regarding the risk of iron overload associated with long-term IV iron therapy, particularly among patients undergoing hemodialysis who require repetitive iron dosing for extended periods of time [18, 19]. Consequently, long-term data assessing the potential for increased oxidative stress and tissue iron deposition in this patient population are needed. In our exploratory substudies, there were no significant differences in biomarkers of oxidative stress or inflammation with ferumoxytol or iron sucrose, and cardiac iron content on MRI was not increased with either IV iron therapy, although LIC was increased in both groups; the clinical relevance of this is unclear. 

In conclusion, as-needed, long-term administration of ferumoxytol demonstrated noninferior efficacy to iron sucrose for the treatment of IDA in patients with CKD undergoing hemodialysis with regard to increasing and maintaining hemoglobin levels within a desired range. The safety profiles of the two agents also appeared to be similar, with no unexpected safety signals emerging during the study. 

## Funding 

The FACT study was funded by AMAG Pharmaceuticals, Inc. Sheridan Henness, PhD, and Sarah Greig, PhD, of inScience Communications, Springer Healthcare, provided medical writing support funded by AMAG Pharmaceuticals, Inc. 

## Conflict of interest 

Iain C. Macdougall has received speakers’ fees, honoraria, and consultancy fees from several ESA and IV iron manufacturers, including Affymax, AMAG, Amgen, Ortho Biotech, Pharmacosmos, Roche, Takeda, and Vifor Pharma. William E. Strauss, Naomi V. Dahl, Kristine Bernard, and Zhu Li are employees of AMAG Pharmaceuticals, Inc. and hold equity in the company. 

**Table 1.** Patient demographics and baseline characteristics (intention-to-treat population).

Characteristic	Ferumoxytol (n = 196)	Iron sucrose (n = 97)	Total (N = 293)
Age, years			
Mean (SD)	59.3 (14.1)	57.6 (13.6)	58.8 (14.0)
Median (range)	59 (24 – 92)	58 (26 – 85)	58 (24 – 92)
Sex, n (%)			
Male	114 (58.2)	57 (58.8)	171 (58.4)
Female	82 (41.8)	40 (41.2)	122 (41.6)
Race, n (%)			
White	101 (51.5)	47 (48.5)	148 (50.5)
Black/African American	62 (31.6)	26 (26.8)	88 (30.0)
Asian	15 (7.7)	13 (13.4)	28 (9.6)
American Indian/Alaskan Native	10 (5.1)	4 (4.1)	14 (4.8)
Other/multiracial	6 (3.1)	4 (4.1)	10 (3.4)
Native Hawaiian/other Pacific Islander	2 (1.0)	3 (3.1)	5 (1.7)
Ethnicity, n (%)			
Hispanic and/or Latino	68 (34.7)	40 (41.2)	108 (36.9)
Height, cm			
Mean (SD)	168.3 (10.5)	167.2 (10.4)	167.9 (10.5)
Median (range)	168 (142 – 196)	168 (144 – 196)	168 (142 – 196)
Weight, kg			
Mean (SD)	86.8 (23.4)	83.2 (21.0)	85.6 (22.75)
Median (range)	84 (40 – 186)	81 (43 – 149)	83 (40 – 186)

SD = standard deviation.

**Table 2.** Summary of AEs in the safety population (N = 293).

AE category	Ferumoxytol (n = 196)		Iron sucrose (n = 97)	
	Events	Patients, n (%)	Events	Patients, n (%)
All TEAEs^a^	1073	158 (80.6)	612	81 (83.5)
Treatment-related TEAEs	14	9 (4.6)	4	4 (4.1)
SAEs	259	93 (47.4)	174	49 (50.5)
Treatment-related SAEs^b^	0	0 (0.0)	0	0 (0.0)
AEs of special interest^c^	30	25 (12.8)	40	26 (26.8)
Cardiovascular AEs^d^	49	29 (14.8)	44	25 (25.8)
AEs resulting in drug discontinuation (temporary)	2	2 (1.0)	9	3 (3.1)
AEs resulting in drug discontinuation (permanent)	11	8 (4.1)	0	0 (0.0)
AEs resulting in study discontinuation	30	20 (10.2)	7	7 (7.2)
Death^e^	22	15 (7.7)	7	6 (6.2)

^a^TEAEs-AEs that occur following randomization and treatment with study drug, i.e., not during screening period. ^b^AEs that are considered related to study drug by the site and investigator. ^c^Including hypotension and hypersensitivity. A hypotension AE of special interest was an AE occurring on the day of dosing and defined as moderate, severe, or life-threatening hypotension or hypotension leading to death; decrease in systolic blood pressure from predose baseline of ≥ 30% during the 30-minute postdose observation period; or lesser reductions in blood pressure associated with symptoms. A hypersensitivity AE of special interest was an AE occurring within 48 hours postdose and defined as moderate to severe local or generalized responses that follow exposure to a potential allergen. ^d^Including myocardial infarction, heart failure, moderate to severe hypertension, and hospitalization due to any cardiovascular cause. ^e^All deaths were reported as unrelated to the study drug by the investigator. AE = adverse event; SAE = serious adverse event; TEAE = treatment-emergent adverse event.

## Supplemental material

Supplemental material Supplemental Tables and Figures
