# Supplementary material for: Ferumoxytol for iron deficiency anemia in patients undergoing hemodialysis. The FACT randomized controlled trial
Source: Clin Nephrol. 2019 Feb 25;91(4):237–45. doi: 10.5414/CN109512 (PMC6434426; doi:10.5414/CN109512)

Supplemental Material

Supplemental Table 1. Changes in oxidative stress/inflammation biomarkers from baseline to week 5 in treatment period 1 (oxidative stress substudy evaluable population).

	**Ferumoxytol**		**Iron sucrose**	
**Time point**	**Parameter**	**Change from baseline**	**Parameter**	**Change from baseline**
**Protein carbonyl content**				
Baseline	0.4 (0.20) [48]	—	0.4 (0.22) [37]	—
Week 5	0.4 (0.34) [54]	0.0 (0.26) [47]	0.4 (0.26) [38]	0.0 (0.25) [36]
**Monocyte chemoattractant protein-1**				
Baseline	319.8 (118.49) [55]	—	347.9 (140.94) [37]	—
Week 5	340.1 (130.38) [54]	26.2 (107.04) [54]	408.1 (469.19) [38]	70.3 (470.76) [36]
**Neutrophil gelatinase-associated lipoprotein**				
Baseline	950.1 (370.23) [57]	—	982.0 (339.89) [37]	—
Week 5	936.7 (289.31) [57]	–13.3 (260.77) [57]	947.5 (368.89) [35]	–31.7 (220.45) [35]
**High-sensitivity interleukin-6**				
Baseline	4.6 (4.34) [55]	—	5.9 (6.15) [37]	—
Week 5	5.1 (5.12) [54]	0.5 (3.90) [54]	11.1 (21.37) [39]	4.9 (19.43) [37]

Data are shown as mean (standard deviation) [n].

Supplemental Table 2. Changes in cardiac and hepatic iron content from baseline to 6 and 12 months (magnetic resonance imaging substudy evaluable population).

	**Ferumoxytol**		**Iron sucrose**	
**Time point**	**Parameter**	**Change from baseline**	**Parameter**	**Change from baseline**
**Cardiac T2*, msec**				
Baseline	37.48 (8.409) [15]	—	35.54 (3.615) [10]	—
6 Months	35.61 (4.578) [14]	–1.90 (9.317) [14]	34.66 (4.374) [9]	–1.04 (6.459) [9]
12 Months	32.67 (4.695) [9]	–2.74 (7.000) [9]	36.06 (4.784) [8]	0.56 (6.547) [8]
**Hepatic T2*, msec**				
Baseline	2.90 (1.407) [15]	—	2.34 (0.934) [10]	—
6 Months	16.30 (6.969) [14]	13.33 (6.966) [14]	6.33 (2.412) [9]	4.05 (2.027) [9]
12 Months	17.43 (9.468) [11]	14.21 (9.405) [11]	5.35 (2.532) [8]	2.95 (2.129) [8]

Data are shown as mean (standard deviation) [n].

Supplemental Figure 1. CONSORT flow diagram

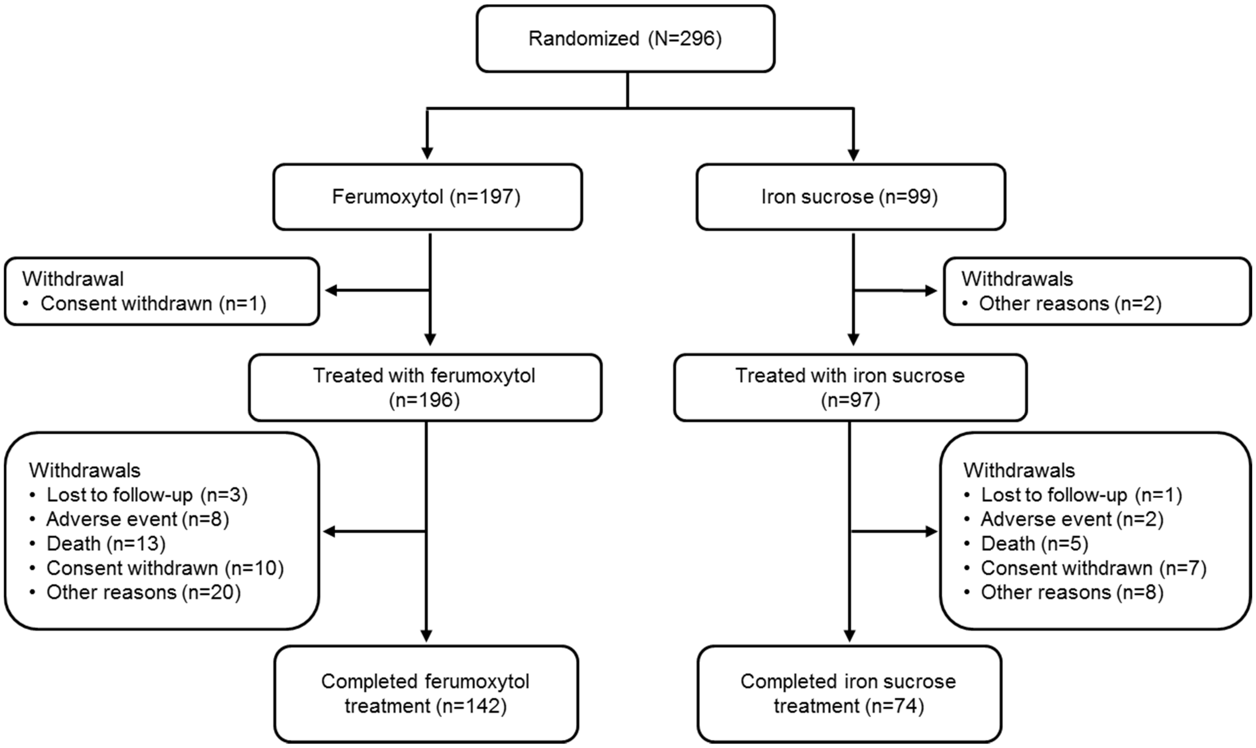

Supplement: Supplemental material [file clinnephrol-91-237-S01.pdf]
